# Incidence and Predictors of Tuberculosis among HIV Positive Children at University of Gondar Referral Hospital, Northwest Ethiopia: A Retrospective Follow-Up Study

**DOI:** 10.1155/2015/307810

**Published:** 2015-05-26

**Authors:** Sualiha Gebeyaw Ayalaw, Kefyalew Addis Alene, Akilew Awoke Adane

**Affiliations:** ^1^Addis Ababa Health Bureau, Addis Ababa, Ethiopia; ^2^Department of Epidemiology and Biostatistics, Institute of Public Health, College of Medicine and Health Sciences, University of Gondar, P.O. Box 196, Gondar, Ethiopia

## Abstract

*Background.* The aim of this study was to determine the incidence of tuberculosis and its predictors among HIV positive children. *Methods.* A six-year retrospective follow-up study was conducted among HIV infected children aged less than 15 years. Life table was used to estimate the cumulative probability of tuberculosis free survival. Cox proportional hazards model was used to identify predictors of tuberculosis. *Results.* A total of 271 HIV positive children were followed for six years and produced 1100.50 person-years of observation. During the follow-up period 52 new TB cases occurred. The overall incidence density of TB was 4.9 per 100 PY. Inappropriate vaccination [AHR: 8.03 (95% CI; 4.61–13.97)], ambulatory functional status [AHR: 1.99 (95% CI; 1.04–3.81)], and having baseline anemia [AHR: 2.23 (95% CI; 1.19–4.15)] were important predictors of time to TB occurrence. *Conclusion.* TB incidence rate was high. Early diagnosis and treatment of anemia and strengthening immunization program would reduce the risk of TB occurrence.

## 1. Background 

Tuberculosis (TB) is a leading cause of morbidity and mortality among HIV infected children. Worldwide, there are approximately nine million new TB cases each year and 13% coinfected with HIV [1, 2]. In 2011, there were estimated 9.2 million new cases of TB, with ten percent of those occurring in children, almost one million new pediatric cases each year [3–5]. TB is among the top ten causes of death among children [6]. One of three HIV coinfected patients dies because of TB and the disease will become worse if it was left untreated [7]. TB accounts for 26% of AIDS-related deaths, of which 99% occur in developing countries [8]. In resource limited countries, TB is the most common opportunistic infection in children with HIV [9–12].

Several studies in Africa have shown that the incidence of TB among HIV positive children ranges from 1 to 9.9 per 100 PY [13–17].

In previous studies, severe wasting, severe immune suppression, anemia, and WHO stage IV were all independently associated with a higher risk of TB. In addition, the use of antiretroviral drugs for more than 180 days reduced the risk of TB [13]. Early initiation of ART (particularly before 12 weeks of age) in HIV infected infants regardless of clinical or CD4 count criteria significantly reduces mortality and TB incidence. Isoniazid preventive therapy (IPT) for TB-exposed infants is an important additional TB prevention strategy [18].

In Ethiopia, childhood TB is still a major cause of hospital admission and death [13]. In 2011, the number of new HIV infections among children was 13 000, 19% of eligible children younger than 15 years old receiving antiretroviral therapy [2]. But studies on various aspects of childhood tuberculosis are rare. Hence, studying the incidence of tuberculosis and its predictors among HIV positive children will have a great importance for the health care system in making appropriate adjustments and allocating resources as a solution.

## 2. Methods

### 2.1. Study Design and Setting

A retrospective follow-up study was conducted in August 2013 at the University of Gondar Referral Hospital pediatrics HIV care clinic, which is located in Northwest Ethiopia. The hospital is the only tertiary hospital located in the historical city of Gondar, serving for more than five million people. During the study period, there were a total of 907 children ever enrolled into pediatrics chronic HIV care and follow-up clinic, 597 ever started ART, and the rest were pre-ART.

### 2.2. Definition of Tuberculosis

In the study setting, TB is diagnosed using chest radiology, fine needle aspiration, and cytology with very high clinical suspicion. When a child is diagnosed with active TB, the treatment is given according to the national TB treatment guideline. The event of this study was new occurrence of TB, which is defined as occurrence of TB in HIV infected children during the follow-up period at any time after enrollment to pediatrics HIV care clinic. Children, who were lost, died, or transferred out or did not develop the events until the last visit were considered as censored. In this study, severe immunodeficiency was defined as CD4 count below the threshold according to the child's age; for infants CD4 <1500/mm^3^ (<25%), 12–35 months <750 mm^3^ (<20%), 36–59 months <350 mm^3^ (<15%), and five years and above <200/mm^3^ (<15%). Those HIV positive children above the threshold were classified as not severe immunodeficiency.

### 2.3. Inclusion and Exclusion Criteria

**Figure 1:**
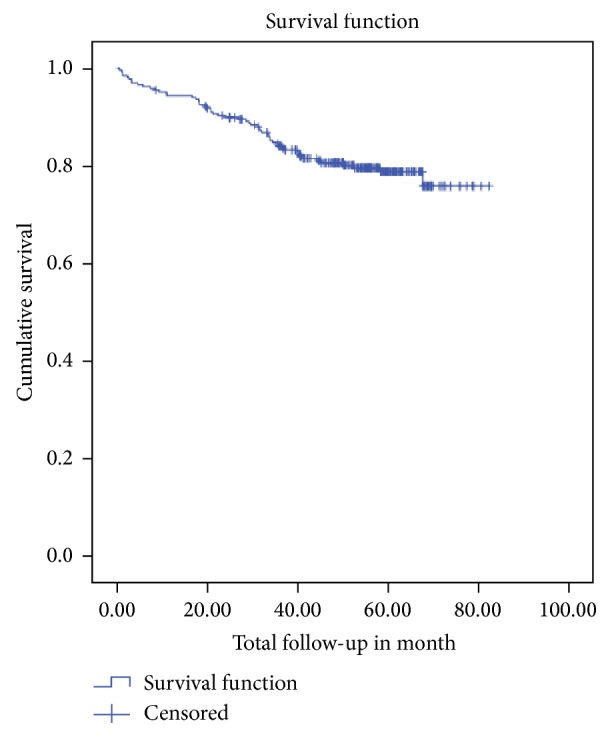
TB free survival proportion of HIV positive children at pediatrics HIV care clinic of University of Gondar Referral Hospital, from September 2006 to August 2013.

All HIV positive children below 15 years of age and newly enrolled into pediatric chronic HIV care clinic at the University of Gondar Referral Hospital from September 2006 to August 2010 were included in this study (Figure 1). Those HIV positive children who started anti-TB treatment at the beginning of the follow-up and those with incomplete baseline information such as CD4 count and hemoglobin (Hgb) level were excluded from the study. Finally, two hundred seventy-one children who fulfilled the inclusion criteria were included in this study.

### 2.4. Data Collection

All available information on patient registration book was checked and an appropriate data extraction tool was prepared. Then, data were extracted from patients' registration book by two health professionals who had ART training and have been working in HIV care clinic.

### 2.5. Data Analysis

Data were entered and cleaned using EPI INFO version 3.5.3 and exported to SPSS version 20 for analysis. Nutritional status was assessed using Anthro plus software. Summary statistics and incidence density rate were calculated. Life table was used to estimate the cumulative probability of TB free survival and Kaplan-meier to estimate the median TB free survival time. Bivariate and multivariate Cox proportional hazards model was used to identify predictors of time to TB occurrence. Adjusted Hazard Ratio (AHR) with 95% confidence intervals (CI) was computed and statistical significance was considered at *P* value ≤ 0.05. The necessary assumptions for Cox proportion hazard model were checked using the Schoen field residual test.

## 3. Ethical Considerations 

Ethical clearance was obtained from the institutional review board of the University of Gondar. Formal letter of permission was obtained from the chief executive officer of the hospital. The Pediatrics ART head gave the consent for extracting data from records. Patients' names and identification numbers were not extracted to ensure confidentiality of patient information.

## 4. Results

### 4.1. Sociodemographic Characteristics of the Study Participants

Two hundred seventy-one HIV infected children were included in the analysis. The mean age was 6.42 (±3.53 SD) years and 41% of them were under 5 years. Slightly more than half (51.7%) of them were females and about 85% of them were from urban areas. One in every eight (12.5%) children was double orphan, who lost both of their partners, and one-third of them were living with more than 5 family sizes (Table 1).

### 4.2. Baseline Clinical Characteristics of Study Participants

Slightly more than half (53.9%) of HIV positive children had a baseline CD4 count of 350 and above. About 16% of them were anemic during enrolment. The baseline median CD4 and Hgb level were 388 (IQR; 193–716) and 12 (IQR; 11.3–13), respectively. During their follow-up, Co-Trimoxazole Preventive Therapy (CPT) was provided nearly for all (97.8%) of patients and about 52% of them received Isoniazid (INH) prophylaxis. Nearly two-third (62%) of them were stunted. During enrolment, about 55%, 37.3%, and 28.1% HIV positive children had functional status of ambulatory, working, and being bedridden, respectively. Nearly one-fifth (16.6%) had past history of TB treatment, and about 11% of them defaulted.

### 4.3. Tuberculosis Incidence Rate

Two hundred seventy-one study subjects were followed up for different periods ranging from one month to six years which resulted in 1100.49 person-years of observation. The mean follow-up time was 48.73 months (SD ± 17.87). Within the follow-up period, one-fifth (19.9%) of new tuberculosis cases were observed and 11% were transferred out. The overall TB incidence density rate was 4.9 per 100 PY.

Nearly three-quarters (70.3%) were pulmonary tuberculosis. Similar proportions (70.3%) of Tb cases were on ART. The cumulative probability of TB free survival at the end of 6 months was 0.96; at the end of a year, 0.94; and that of survival at the end of three years was 0.84 and at the end of six years was 0.76. The median survival time from enrolment to TB occurrence was 72 months.

### 4.4. Predictors of Time to TB Occurrence

In bivariate Cox-regression analysis, paternal orphans, baseline WHO clinical stage IV, and having baseline anemia and being ambulatory and inappropriately vaccinated were significantly associated with incidence of TB among HIV positive children. However, in multivariate Cox-regression analysis, being inappropriately vaccinated and having baseline anemia and ambulatory functional status remained significant predictors of TB occurrence.

Those HIV infected children who had inappropriate vaccination were about 8 times (AHR 8.03 (95% CI; 4.61–13.97)) higher risk of developing TB at any time as compared to the counterpart. Those HIV infected children who had baseline anima are 2.2 (AHR 2.23 (95% CI; 1.19–4.15)) times higher risk of developing TB at any time as compared to those HIV infected children who had no baseline anemia. HIV infected children who had ambulatory function status at enrollment were at 1.9 (AHR 1.99 (95% CI; 1.04–3.81)) times higher risk of developing TB compared to those who were working (Table 2).

## 5. Discussion

This study revealed that the overall incidence density rate of TB among HIV positive children at University of Gondar Referral Hospital was 4.9 PY. It was similar with the finding from a cohort study in Tanzania (5.2 cases per 100 PY) [13]. But, this finding is higher than that of studies done in Kenya and South Africa which were 1.4 and 1.0 per 100 PY, respectively [14, 15]. The discrepancies in incidence rate may be due to the difference in follow-up period of the studies and the difference in the overall burden of TB in the general population. But the rate was lower than the finding from a cohort study in Felege Hiwot Referral Hospital (Northern Ethiopia) which was 9.9 per 100 PY (7 cases out of 56 children followed up for 70.50 PY) [19]. This may be due to the difference in the study population (mixed adult and pediatric).

This study found that HIV positive children with baseline anemia had 2.23 times higher risk of developing TB as compared to nonanemic children which is similar to the studies done in Tanzania and Northern Ethiopia [13, 19]. This could be due to the fact that children were affected by infections when their Hgb level is lower than 12 g/dL. As a result, they become at higher risk for TB.

This study also found that patients with baseline ambulatory functional status had 2 times higher risk of developing TB as compared to working functional status. This finding is similar to the study done in Northern Ethiopia [20]. This could be due to the fact that patients lose their functional status as a result of many infectious diseases when they are anemic.

This study also revealed that patients who were not appropriately vaccinated had 8.03 times higher risk of developing TB as compared to vaccinated children. Similar finding in Tanzania showed that BCG scar was associated with a reduced risk of TB, as research showed BCG vaccination considerably reduced the risk of TB, both among individuals with and without HIV infection [21].

Unlike the previous studies, the current study found that baseline clinical factors like immune suppression, WHO stage, ART status, and nutritional status were not associated with occurrence of TB. For instance, a study in Tanzania reported severe wasting (RR 1.8, 95% CI 1.3–2.5), severe immune suppression (RR 2.6, 95% CI 1.8–3.8), and WHO stage IV (RR 4.5, 95% CI 2.4–8.5) were all independently associated with a higher risk of TB [21]. In addition, the use of antiretroviral drugs for more than 180 days reduced the risk of TB by 70% (RR 0.3, 95% CI 0.2–0.4) [21]. The discrepancy might be due to methodology, study setting, and time variations.

The retrospective nature of the study was one of the limitations of this study. As a result, some of the important predictors which had a significant association with the TB occurrence in other studies like income were not included in this study.

## 6. Conclusion

Incidence of TB was high among HIV infected children, especially after the first six months of enrolment in HIV care. The baseline anemia, ambulatory functional status, and those who were not appropriately vaccinated were significantly associated with the incidence of tuberculosis. Therefore, early diagnosis and treatment of anemia and strengthening immunization program are recommended to reduce the risk of TB occurrence among HIV infected children.

## Figures and Tables

**Table 1:** Sociodemographic characteristics of HIV positive children at pediatrics HIV care clinic of the University of Gondar Referral Hospital, Northwest Ethiopia, from September 2006 to August 2010.

Characteristics	Categories	Frequency	Percent
Sex	Male	131	48.3
	Female	140	51.7

Age	<5	111	41.0
	5–9	102	37.6
	10–14	58	21.4

Residence	Urban	231	85.2
	Rural	40	14.8

Parental status	Both parents alive	138	50.9
	Paternal orphan	56	20.7
	Maternal orphan	34	12.5
	Double orphan	34	12.5
	Not recorded	9	3.3

Family size	≤2	29	10.7
	3-4	150	55.4
	≥5	92	33.9

Care givers	Parents	203	74.9
	Sibling	24	8.9
	Grand parents	26	9.6
	Guardians	12	4.4
	Orphanage centers	6	2.2

**Table 2:** Cox-regression analysis of predictors of incidence of TB among HIV infected children at University of Gondar Referral Hospital (*n* = 271).

Variables	TB incidence		CHR (95% CI)	AHR (95% CI)
	Event	Censored		
Sex				
Male	28	103	1	
Female	26	114	0.84 (0.49–1.43)	
Age				
<5	18	93	0.87 (0.41–1.84)	
5–9	25	77	1.33 (0.65–2.69)	
10–14	11	47	1	
Residence				
Urban	44	187	1	
Rural	10	30	1.58 (0.79–3.15)	
Family size				
≤2	6	23	1	
3-4	31	119	0.94 (0.39–2.26)	
≥5	17	75	0.82 (0.32–2.07)	
Parental status				
Both parents a live	22	116	1	
Paternal orphan	17	39	2.05 (1.09–3.87)	
Maternal orphan	7	27	1.26 (0.54–2.95)	
Double orphan	5	29	0.90 (0.34–2.38)	
Not recorded	3	6	2.27 (0.68–7.58)	
WHO clinical stage				
I	10	73	1	
II	13	59	1.49 (0.65–3.41)	
III	23	71	2.06 (0.98–4.33)	
IV	8	14	3.66 (1.44–9.29)	
Anemia				
Anemia	15	27	2.47 (1.36–4.49)	2.23 (1.19–4.15)
No Anemia	39	190	1	
Immunodeficiency				
Severe	20	77	1.08 (0.62–1.87)	
Not severe	34	140	1	
ART at initial				
Yes	22	90	1.10 (0.64–1.91)	
No	32	127	1	
IPT				
Yes	28	114	1.09 (0.64–1.85)	
No	26	118	1	
CPT				
Yes	53	212	0.82 (0.11–5.96)	
No	1	5	1	
Past TB treatment history				
Yes	11	34	0.82 (0.11–5.96)	
No	43	183	1	
Functional status				
Working	13	88	1	
Ambulatory	37	111	2.21 (1.17–4.19)	1.99 (1.04–3.81)
Bedridden	4	18	1.50 (0.49–4.61)	0.92 (0.29–2.92)
Vaccination status				
Vaccinated	22	174	1	
Not appropriately vaccinated	31	22	7.86 (4.54–13.62)	8.03 (4.61–13.97)
Not recorded	1	21	0.36 (0.05–2.64)	0.42 (0.06–3.15)
Nutritional status				
Normal	19	84	1	
Stunted	35	133	1.07 (0.61–1.87)	

COR = Crude hazard ratio, AOR = Adjusted Hazard Ratio, and CI = confidence interval.
